# Association study of the functional Catechol-O-Methyltranferase (COMT) Val^158^Met polymorphism on executive cognitive function in a Thai sample

**DOI:** 10.7150/ijms.35789

**Published:** 2019-09-20

**Authors:** Bupachad Khanthiyong, Samur Thanoi, Gavin P. Reynolds, Sutisa Nudmamud-Thanoi

**Affiliations:** 1 Department of Anatomy, Faculty of Medical Science, Naresuan University, Phitsanulok, Thailand; 2 Centre of Excellence in Medical Biotechnology, Faculty of Medical Science, Naresuan University, Phitsanulok, Thailand; 3 Biomolecular Sciences Research Centre, Sheffield Hallam University, Sheffield, United Kingdom

**Keywords:** Executive function, Catechol-O-Methyltranferase (COMT), Val^158^Met, Wisconsin Card Sorting Test (WCST), Single Nucleotide Polymorphism (SNP)

## Abstract

Catechol-O-Methyltranferase (COMT) plays a crucial role in the removal of cortical dopamine and is strongly implicated in human executive function. Numerous studies have reported associations of the COMT Val158Met (rs4680) polymorphism with executive function in healthy subjects. However, little work has investigated this in the Thai population and the relationship of age and education with this association remains unclear. Therefore, this study was designed to investigate the association of this polymorphism of the COMT gene with executive cognitive brain function in healthy subjects and the relationship with age and education. The Wisconsin Card Sorting Test (WCST) was performed to assess executive function in 254 healthy Thai subjects (aged 20-72 years). The results showed a significant association of rs4680 with executive function, in which Val/Met heterozygotes demonstrated better cognitive set shifting performance. Moreover, Met allele carriers showed a significantly stronger effect in the categories completed score than did Val homozygotes. Furthermore, age and education also showed a significant association with COMT genotype and WCST. These results revealed that executive cognitive function is associated with COMT genotype and influenced by age and/or education level in a Thai sample.

## Introduction

Executive function is a higher cognitive ability that uses previous experiences and new information to regulate and manage thoughts and actions for successful goal-directed behavior. Executive function processes include planning or organizing, working memory, focus or attention, problem-solving, verbal reasoning, decision-making, cognitive set shifting, self-monitoring and regulation of emotion 1,2. These complex behaviors are mediated by the prefrontal cortex (PFC) and other brain regions. Currently, various tasks have been used to assess executive function including Trail Making Tests A and B, digit span test, Stroop test, word-fluency test and Wisconsin Card Sorting Test (WCST). WCST is one of the most popular tasks for measurement of prefrontal cortex function 3.

Dopamine (DA) has been reported to be an important neurotransmitter related to executive function 4. Catechol-O-Methyltransferase (COMT) is one enzyme responsible for the degradation of dopamine and regulates the concentration of dopamine, and hence its biological action, in the cortex. Genetic polymorphisms affecting expression or regulation of COMT might therefore influence executive function. A functional single nucleotide polymorphism (SNP) of COMT is Val^158^Met (rs4680) leading to the alteration of enzyme activity; the Met allele produces COMT with a low activity which in turn reduces the degradation rate and increases cortical DA. On the other hand, the higher enzymatic activity of the Val allele results in decreased DA activity 5,6. At present, numerous studies have reported that this COMT SNP is associated with executive function in healthy subjects, as well as in subjects with schizophrenia, bipolar disorder, and attention deficit hyperactivity disorder among others 7,8,9. Numerous studies report that the Met allele is associated with better performance in executive function when compared with the Val allele in healthy subjects 10,11. Barnett and coworkers 12 identified a relationship of rs4680 with perseverative errors on the WCST in healthy subjects and schizophrenia. However, healthy volunteers, but not schizophrenic patients, with the Met/Met genotype showed better WCST performance. Moreover, the Met/Met genotype, related to higher PFC DA, is associated with the best performance in attention tasks in adult subjects whereas adolescents with Val/Met genotype showed a better attention than Met/Met and Val/Val genotypes 13.However, a meta-analysis has shown no significant effect, although this might relate to differences between samples studied 14. There also appear to be suggestions of an age effect 15,16, while education level was also reported to be one factor affecting executive ability 17,18. However, these issues have been little studied in Thailand and remain unclear. Therefore, this study was designed to investigate the association of the rs4680 polymorphism of the COMT gene with executive cognitive brain function in healthy volunteers and the relationship with age and education.

## Materials and methods

### Subjects

All subjects were Thai aged between 20-72 years and provided written informed consent prior to the study. Level of education was determined and subjects assigned to two groups: those receiving no more than primary education, and those who had received secondary and, for some, tertiary education. Subjects with abnormal mental health evaluated by the Thai Mental Health Indicator (TMHI-55) were excluded from this study. The Mini-Mental State Examination (MMSE) was also used to exclude subjects with dementia. All experimental protocols in this study were approved by the Human Ethics Committee of Naresuan University (COA No. 553/2017).

### Single nucleotide polymorphism (SNP) study

The blood sample and DNA extraction was performed using 2 different methods. The blood samples from the cubital vein were collected in EDTA blood collection tube and the genomic DNA was extracted from blood leukocytes by using Trizol LS reagent following by the manufacturing instruction. The fingertip blood samples were collected on FTA cards and the DNA extraction was performed following a previous report 19. The genotyping of COMT Val^158^Met SNP (rs4680) was conducted by polymerase chain reaction restriction fragment length polymorphism (PCR-RFLP) method. The amplification of 100 ng DNA template by PCR in a total reaction volume of 25 µl using forward primer 5'-TACTGTGGCTACTCAGCTGTGC-3' and reverse primer 5'-GTGAACGTGGTGTGAACACC-3'^20^. PCR conditions were performed as following: 1) predenaturation at 95°C for 2 min and 45 cycles of denaturation at 95°C for 30 sec, 2) annealing at 65°C for 30 sec, 3) extension at 72°C for 30 sec, and 4) the last cycle of PCR were performed at 72 °C for 5 min. The 236 bp PCR products were digested with restriction enzyme: Hsp92II (promega) and incubated at 37˚C for 2 hr. The complete digestion produces 4 fragments of size 114 bp and/or 96 bp, 54 bp, 44 bp, and 24 bp in which 114 bp represents Val/Val homozygotes, both 114 bp and 96 bp are Val/Met heterozygotes, and 96 bp Val/Val homozygotes. The fragments were separated using 4% agarose gel electrophoresis and visualized by ethidium bromide staining.

### Executive function test

The Wisconsin Card Sorting Test (WCST) is widely used to test for frontal cortex function in clinical and research contexts 14. In this study, the subjects were tested by computer-based WCST (Inquisit 3.0.6.0) to assess executive function. Four stimulus cards and 128 response cards were used for the assessment. The response cards contain 3 different dimensions of colors (red, blue, yellow or green), numbers of objects (1,2,3 or 4) and forms (crosses, circles, triangles or stars). The sorting rule is based on color, number, or form but not given to the subject. For this WCST, 4 stimulus cards are shown on the screen of a laptop computer along with a single response card. At the beginning, the instruction of the test was given to subject and subject has to select the correct card matching the response card according to the sorting rule. After matching, the subject is informed of the result (right or wrong). After 4 consecutive correct matches, one completed category, the sorting rule shifts to the next sorting rule without prior warning. This test continues until the subject has either completed 6 categories of 3 different sorting rules or all 128 cards have been used 21.WCST raw score was analyzed and reflected different aspects of executive function as follows 22:

- The number of categories completed was determined using the score range between 1 and 6, reflecting cognitive set shifting.
- Trials to complete the first category was determined the ability to formulate a logical concept with the score range between 0 and 128, reflecting initial conceptualization.
- The perseverative errors were used to measure the inability to correct the respond due to ignorance of relevant stimuli, reflecting cognitive inflexibility.
- The percentage of total corrects: the total number of correct response cards multiply 100 and divided by total cards, reflecting initial conceptualization and attention.
- The percentage of total errors: the total number of incorrect responses cards multiply 100 and divided by total cards, reflecting nonspecific cognitive impairment.

### Statistical analysis

SPSS software (IBM SPSS statistics version 23) was used for analyses employing the Pearson Chi-squared test, univariate general linear model, independent t-test, Spearman rank correlation. The significance level was considered at p≤0.05.

## Results

### Demographic data and effects on genotype

Subjects comprised 110 males and 144 females with a mean age of 46.41±18.32 years (range, 20-72 years). Their demographic data including education, age and sex are described according to genotype in table 1. Age however differed between both sexes (females 43.91±18.80 males 49.68±17.22: t=2.54; p=0.012) and education (t=16.53; p<0.001) categories, although there was no significant relationship between sex and education level (χ^2^=2.44; p=0.118).

The distribution of genotype shown in table 1 is consistent with proportions expected under Hardy-Weinberg equilibrium (p=0.078 by χ^2^ test).No significant difference in age was found between genotypes, although the Val/Val group showed a slightly higher mean age. There were no significant differences in genotype between male and female subjects or in relation to level of education.

### Demographic effects on WCST results

All measures except the first category were significantly correlated with age using Spearman rank correlation (Table 2). An effect of educational level on WCST performance was found in % total correct, % total errors and categories completed as shown in table 3. Sex was found to have a significant effect only on the WCST subscale % total correct (p=0.049). Age was included as a covariate in further analyses.

### COMT genotype effect on executive function

A significant association between COMT genotype and WCST performance was found for categories completed, in which Val/Met heterozygotes showed improved performance (Table 4). Analysing the results using a two-genotype dominant model (Table 5) showed a somewhat stronger effect in which Met allele carriers showed better performance that Val/Val homozygotes in the categories completed. A recessive model showed no significant differences in any WCST measures (data not shown).

## Discussion

In this study of a sample of healthy subjects from the Thai population, we find an association of measure of cognitive function from the WCST with the rs4680 Val/Met COMT polymorphism, in which carriage of the Met allele is significantly associated with better cognitive set shifting. The study also demonstrated that age and education both influence WCST performance. The two are closely related, reflecting the rapid increase in access to education in Thailand so that more younger people have education beyond a primary level. This relationship makes it difficult to determine which may have a greater influence on cognitive performance as a decline with age is reported 16, as is a relationship with years of education 17,18. We chose to use age, functioning also as a proxy for the effect of education, as a covariate in statistical analyses of the genetic effect here. The polymorphism was associated with one measures of cognitive function obtained from the WCST. The Val/Met genotype and Met allele carriers demonstrated an increased number of categories completed, which is a measure of cognitive set shifting, one aspect of executive function. There is a strong body of evidence indicating that the Met allele is associated with better executive function than the Val allele, although these findings primarily relate to scores of perseverative errors on the WCST 23,24, which we find not to be significantly changed in our study. However, our results showed a difference in categories completed but not in either total correct or total error which were reported previously 23,24. Ethnicity may affect the results even though the study has done in the healthy subjects; all subjects in this study are Thai while the study of Malhotra et al. 23 have only 3 Asians. Our study has also assessed both male and female subjects but the study of Caldu et al. 24 was only done in females. One of the factors may affecting on the WCST is age; the mean age of the subjects in our study is different from those previous studies.

It is well-established that rs4680 is functionally related to COMT enzyme activity, in which the highest activity is associated with the Val/Val genotype, and the lowest with Met/Met 5,6. This results in differences in the neurotransmitter activity of dopamine in the frontal cortex. Furthermore, a recent study has found that COMT Val carriers have a thinner cortex in prefrontal, parietal, and posterior cingulate cortices than COMT Met carriers independent of age, indicating effects on cortical structure, and that genotype and cortical thickness influenced executive function 25.

There are several limitations to our study. In an attempt to obtain a sample approximately representative of the population, the sample covers a range of the population of varying ages and educational background. We have been unable to distinguish the relative and overlapping effects of these two variables on executive function; future studies could address this by selecting a large sample with a more limited age range. Such factors add to the variance in the WCST results and may have contributed to the limited effect of genotype, which did not significantly influence perseverative errors as might be expected. The sample size was not large which limited the opportunity to subdivide the sample further in order, for example, to study the relative effect of sex.

Nevertheless, these findings indicate that executive cognitive function is associated with COMT genotype and influenced by age and/or education level in a healthy Thai sample.

## Figures and Tables

**Table 1:** Demographic data of the three COMT genotypes

	Val/Val (N=141)	Val/Met (N=89)	Met/Met (N=24)	p-value
Age	47.93±18.12	44.54±18.55	44.42±18.58	0.337
Sex (Female/Male)	81(57.45%)/60(42.55%)	53(59.55%)/36(40.45%)	10(41.67%)/14(58.33%)	0.281
Educational level				
Primary	71 (50.35%)	39 (43.82%)	12 (50%)	0.614
Secondary and tertiary	70 (49.65%)	50 (56.18%)	12 (50%)	

Data were presented as mean±SD by univariate general linear model.

**Table 2:** The correlation of WCST scores with age in healthy volunteers

WCST (N=254)	Correlation Coefficient	p (2-tailed)
%Total correct	-0.167	0.007**
%Total error	0.218	0.008**
1st category completed	0.105	0.095
Categories completed	-0.134	0.032*
Perseverative error	0.214	0.001***

Data were analyzed by Spearman rank correlation. Value were considered significant at *p≤0.05, **p≤0.01, ***p≤0.001.

**Table 3:** Association of WCST scores of healthy volunteers with education level

	Educational level		
WCST (N=254)	Primary (N=122)	Secondary/Tertiary (N=132)	p-value
% Total correct	43.53±12.49	48.99±12.49	0.000***
% Total error	56.11±11.55	50.97±12.45	0.001***
1st category completed	12.84±12.61	11.2±12.84	0.304
Categories completed	4.60±1.57	5.03±1.51	0.027*
Perseverative error	3.00±4.19	2.50±5.89	0.440

Data were presented as mean±SD. *p≤0.05 ***p≤0.001 by univariate general linear model.

**Table 4:** WCST scores of healthy volunteers with genotype of rs4680 in COMT gene

	COMT genotype (frequency)		Covariate with age		Covariate with education
WCST (N=254)	Val/Val (N=141)	Val/Met (N=89)	Met/Met (N=24)	p-value	p-value
% Total correct	45.18±12.63	48.35±13.12	46.00±9.48	0.259	0.240
% Total error	54.77±12.63	51.17±12.16	54.04±9.52	0.143	0.130
1^st^ category completed	12.59±13.42	10.97±9.85	12.25±17.51	0.728	0.677
Categories completed	4.58±1.60	5.13±1.49	5.08±1.28	0.034*	0.027*
Perseverative error	3.18±6.18	2.50±3.59	1.04±2.05	0.183	0.170

Data were presented as mean±SD. *p≤0.05 by univariate general linear model as age and education covariate.

**Table 5:** WCST scores of healthy volunteers with Val/Val and Met allele carriers of rs4680 in COMT gene

	COMT genotype (frequency)		Covariate with age	Covariate with education
WCST (N=254)	Val/Val genotype (N=141)	Met allele carriers (N=113)	p-value	p-value
% Total correct	45.18±12.63	47.85±12.43	0.156	0.126
% Total error	54.77±12.63	51.78±11.67	0.094	0.073
1st category completed	12.59±13.42	11.24±11.81	0.507	0.432
Categories completed	4.58±1.60	5.12±1.45	0.009**	0.007**
Perseverative error	3.18±6.18	2.19±3.37	0.173	0.207

Data were presented as mean±SD. **p≤0.01 by univariate general linear model as age and education covariate.

## Abbreviations

COMT: catechol-o-methyltransferase
SNP: single nucleotide polymorphism
WCST: wisconsin card sorting test
PCR: polymerase chain reaction
RFLP: restriction fragment length polymorphism
TMT-AB): tail making test A, B
TMHI-66: Thai mental health indicator
MMSE: mini-mental state examination
HWE: Hardy-Weinberg equilibrium
